# The Complete Plastid Genome of *Lagerstroemia fauriei* and Loss of rpl2 Intron from *Lagerstroemia* (Lythraceae)

**DOI:** 10.1371/journal.pone.0150752

**Published:** 2016-03-07

**Authors:** Cuihua Gu, Luke R. Tembrock, Nels G. Johnson, Mark P. Simmons, Zhiqiang Wu

**Affiliations:** 1 School of Landscape and Architecture, Zhejiang Agriculture and Forestry University, Hangzhou 311300, P.R. China; 2 Department of Biology, Colorado State University, Fort Collins, Colorado, 80523, United States of America; 3 National Institute for Mathematical and Biological Synthesis, University of Tennessee, Knoxville, 37996, Tennessee, United States of America; University of Cambridge, UNITED KINGDOM

## Abstract

*Lagerstroemia* (crape myrtle) is an important plant genus used in ornamental horticulture in temperate regions worldwide. As such, numerous hybrids have been developed. However, DNA sequence resources and genome information for *Lagerstroemia* are limited, hindering evolutionary inferences regarding interspecific relationships. We report the complete plastid genome of *Lagerstroemia fauriei*. To our knowledge, this is the first reported whole plastid genome within Lythraceae. This genome is 152,440 bp in length with 38% GC content and consists of two single-copy regions separated by a pair of 25,793 bp inverted repeats. The large single copy and the small single copy regions span 83,921 bp and 16,933 bp, respectively. The genome contains 129 genes, including 17 located in each inverted repeat. Phylogenetic analysis of genera sampled from Geraniaceae, Myrtaceae, and Onagraceae corroborated the sister relationship between Lythraceae and Onagraceae. The plastid genomes of *L*. *fauriei* and several other Lythraceae species lack the *rpl2* intron, which indicating an early loss of this intron within the Lythraceae lineage. The plastid genome of *L*. *fauriei* provides a much needed genetic resource for further phylogenetic research in *Lagerstroemia* and Lythraceae. Highly variable markers were identified for application in phylogenetic, barcoding and conservation genetic applications.

## Introduction

The Lythraceae include approximately 620 species in 31 genera; most are herbs, with some trees and shrubs adapted to a wide variety of habitats. The four largest genera (*Cuphea*, *Diplusodon*, *Lagerstroemia*, and *Nesaea*) include three-fourths of all species in Lythraceae [1]. The family has been traditionally classified in the order Myrtales and closely allied with the Onagraceae based on morphological, anatomical, and embryological evidence [2,3]. Within the Lythraceae, *Lagerstroemia* (“crape myrtle”) is the most economically important and well-known genus. *Lagerstroemia* comprises about 55 species [4–6] and its center of diversity is in southeast Asia and Australia [7], mainly in tropical and sub-tropical habitats of southern China, Japan, and northeast Australia. Most *Lagerstroemia* species are easily propagated, resistant to multiple pathogens, grow rapidly, and have colorful flowers that open from summer to fall [8]. Given the importance of *Lagerstroemia* as an ornamental, more than 260 cultivars have been created and registered (http://www.usna.usda.gov/Research/Herbarium/Lagerstroemia/index.html). Due to the ornamental and economic value of *Lagerstroemia*, research programs have been initiated to develop hybrid cultivars, study the genetic diversity of cultivars, and evaluate germplasm [9–13]. Molecular tools have been employed to identify *Lagerstroemia* cultivars and interspecific hybrids [14,15]. Despite the development of microsatellite markers and subsequent research in *Lagerstroemia*, no complete chloroplast (plastid) genomes have been described from Lythraceae.

Phylogenomic-related research in Lythraceae is limited. Within the Myrtales, Lythraceae was resolved as sister to Onagraceae using the plastid gene *rbcL* [16]. Within Lythraceae, *Lagerstroemia* and *Duabanga* are supported as sister groups based on *atpB-rbcL*, *psaA-ycf3*, *rbcL*, *trnK-matK*, *trnL-trnF*, and ITS (internal transcribed spacer region of the nuclear genome) data [1,17]. Phylogenetic inferences within *Lagerstroemia* and the Lythraceae could be improved if plastid genomes are made available, potentially providing dozens of valuable molecular markers for further research.

In contrast to huge nuclear genomes, the plastid genome, with uniparental inheritance, has a highly conserved circular DNA arrangement ranging from115 to 165 kb [18,19], and the gene content and gene order are conserved across most land plants [20]. With the development of next-generation sequencing approaches, sequencing whole plastid genomes has become cheaper and faster [21]. To date, more than 900 land-plant species’ completed plastomes can be accessed through the National Center for Biotechnology Information (NCBI) public database [22]. Such genetic resources have provided a useful set of tools for researchers interested in species identification by using DNA barcoding [23], genetic data used for plastid transformation [24], and designing molecular makers for systematic and population studies [25,26]. All of these research areas have benefitted from the conserved sequences and structure as well as the lack of recombination found in plastid genomes to simplify analyses. For example, plastids maintain a positive homologous recombination system [27–30], which enables precise transgene targeting into a specific genome region during transformation. Different plastid loci have been used for evaluating phylogenetic relationships at different taxonomic levels, including the interspecific and intraspecific levels [31]. Recently phylogenomic approaches [32] to study plant relationships have employed complete-plastid-genome sequences for studying phylogenetic relationships.

In an effort to comprehensively understand the organization of the *Lagerstroemia* plastid genome, we present the first complete plastid genome sequence of *L*. *fauriei*, which was generated using Illumina sequencing. The three aims of our study are to: deepen our understanding of the structural diversity of the complete *L*. *fauriei* plastid genome, compare molecular evolutionary patterns of the *L*. *fauriei* plastid genome with other plastid genomes in the Myrtales, and provide a set of genetic resources for future research in *Lagerstroemia* and the Lythraceae.

## Materials and Methods

### Plant materials, DNA extraction and sequencing

Leaves of *L*. *fauriei* were obtained from the nursery of Zhejiang Agriculture and Forestry University (Hangzhou, Zhejiang, China) and preserved in silica gel. Total genomic DNA was extracted from leaves using a cetyl-trimethyl-ammonium-bromide DNA-extraction protocol [33]. Total genomic DNA was used to construct a sequence library following the manufacturer's instructions (Illumina Inc., San Diego, CA). Paired-end (PE) sequencing libraries with an insert size of approximately 300 bp were sequenced on an Illumina HiSeq 2000 sequencer at the Beijing Genomics Institute (BGI) and 30,887,628 clean reads were obtained, each with a read length of 100 bp.

### Plastid genome assembly and annotation

The raw Illumina reads were demultiplexed, trimmed and filtered by quality score with Trimmomatic v0.3 [34] using the following settings: leading: 3, trailing: 3, sliding window: 4:15 and minlen: 50. Then the CLC Genomics Workbench v7 (CLCbio; http://www.clcbio.com) was used to conduct *de novo* assembly of reads from *L*. *fauriei* with the default parameters. The following three separate *de novo* assemblies were made: PE reads, single-end forward reads and single-end reverse reads [22]. These three separate assemblies were then combined into a single assembly. Assembled contigs (≥0.5 kb) with > 100× coverage from the complete CLC assembly were compared to several Myrtales species with completed plastid genomes, including *Oenothera argillicola* (Onagraceae; NC_010358), *Syzygium cumini* (Myrtaceae; GQ870669), and *Eucalyptus aromaphloia* (Myrtaceae; NC_022396). Local BlastN [35] searches were used to match the contigs from the plastid genomes. Based on the conserved features of the plastid genome [19,22], the mapped contigs were orientated onto the related plastid genomes [36] and those separate contigs were connected into a single contig to construct the circular map of the genome using Informax Vector NTI Contig Express 2003 (Invitrogen, Carlsbad, CA). Seven short gaps (≤100 bp) were filled by aligning individual Illumina sequence reads that overlapped at the contig ends. Longer gaps (>100 bp) between contigs were filled by designing primers in flanking regions, conducting PCR amplifications, and closing the gap regions by adding sequence data generated from Sanger sequencing (by BGI).

We designed additional primers (S1 Table) to test for correct sequence assembly. PCR was conducted in 40μl volumes containing 4 μl 10× Taq buffer, 0.8 μl dNTP (10 mM), 0.4μl Taq polymerase (5 U/μl), 0.5ul each primer (20 pmol/ul; all from Sangong Biotech (Shanghai, China)), 0.5 ul DNA template, and 33.3 μl ddH_2_O. The amplification program consisted of an initial heating at 94°C for 5 min, then 32 cycles including denaturation at 94°C for 45 s, annealing at 55°C for 45 s, elongation at 72°C for 2 min, and a final elongation at 72°C for 10 min. After incorporation of the Sanger results, the finished plastid genomes were applied as the reference to map the previously unincorporated short reads in order to iteratively refine the assembly based on evenness of sequence coverage.

DOGMA v1.2 [37] was employed for genome annotation of the protein-coding genes, transfer RNAs (tRNAs) and ribosomal RNAs (rRNAs). To accurately confirm the start and stop codons and the exon-intron boundaries of genes, the draft annotation was subsequently inspected and adjusted manually based on plastomes from a related species, *Syzygium cumini* [36], from the NCBI database. Additionally, both tRNA and rRNA genes were identified by BLASTN searches against the same database of plastomes. Finally, tRNAscan-SE v1.21 [38] was also used to further verify the tRNA genes. The schematic diagram of the plastid genome map was generated using OGDraw [39].

### Comparative plastid genomic analysis

#### Expansion and contraction of four junction regions

Genome-size variation among different photosynthetic species is generally caused by different junctions between the two inverted-repeat regions (IR_A_ and IR_B_) and the two single-copy regions (LSC and SSC) [36]. There are four junctions (J_LA_, J_LB_, J_SA_, and J_SB_) in the plastid genome between the two single copy (LSC and SSC) regions and the two IRs (IR_A_ and IR_B_) [40]. The detailed IR border positions and the adjacent genes among seven Myrtales species plastomes (*Lagerstroemia fauriei*, *Oenothera argillicola*, *Angophora costata*, *Corymbia eximia*, *Eucalyptus aromaphloia*, *Stockwellia quadrifida*, and *Syzygium cumini*) were compared in this study.

#### Survey for loss of the rpl2 intron

In the process of annotation and comparison with other species in the Myrtales, we found that the intron of *rpl2* is absent in the plastome of *L*. *fauriei*. In order to infer the history of this intron loss, we designed a pair of primers (Forward-CAAAACTTCTACCCCAAGCA; Reverse-TCTTCTTCCAAGTGCAGGAT) to amplify the whole *rpl2* region and then applied them to 11 *Lagerstroemia* species and three species (*Cuphea hyssopifolia*, *Punica granatum*, and *Lythrum salicaria*) from other Lythraceae genera, as well as the outgroups *Oenothera albicaulus* and *Catha edulis*. In *L*. *fauriei*, the target *rpl2* fragment without the intron is about 750 bp, whereas it is about 1,400 bp in species containing the intact intron. PCR was used to amplify the *rpl*2 region and the amplicons were run out on 1% agarose gels. Fragment sizes were determined by comparison to DNA size standards [41]. Sanger sequencing of forward and reverse sequence of gene *rpl2* was done for *Cuphea hyssopifolia*, *Punica granatum*, *L*. *salicaria*, *L*. *fauirei*, *L*. *limii* and *Oenothera albicaulus* at the Proteomics and Metabolomics Facility of Colorado State University.

#### Repetitive sequence analysis

Repetitive elements were investigated using two different approaches. In order to avoid redundancy, repeat-sequence analysis was only carried out using just one IR region [42]. Tandem Repeat Finder [43] was used with the minimum-alignment score and maximum-period size set at 50 and 500, respectively, with default parameters for all other search criteria to find small tandem repeats from 15 to 30 bp in length. The numbers of forward, reverse, complementary and palindromic repeats were quantified using the REPuter [44], setting Hamming distance equal to three and minimum repeat size ≥30 bp. Overlapping repeats were merged into one repeat motif where possible. Microsatellites (SSRs) were detected using SSR Hunter v1.3 [45]. We identified SSRs as mononucleotides with ≥ 8 repeats, dinucleotides ≥ 4, trinucleotides ≥ 3, and tetranucleotides and pentanucleotides both ≥ 3.

#### Dot-plot analysis

We compared plastomes of the other six Myrtales species to *L*. *fauriei* with dot-plot analysis using Perl scripts to visualize arrangement recurrences and structural differences in two-dimensional plots (S1 Fig).

#### Informative variables analysis from coding and non-coding regions

To identify divergent regions that may be highly informative for phylogenetic analyses, each region, including CDS (coding regions), introns, and IGS (intergenic regions) from seven Myrtales plastid genomes was individually examined. For the longer genes (>1500 bp), we employed the sliding window method to divide the gene into shorter fragments to detect the most informative portions by using a 1000 bp sliding window and 500 bp increments. These regions were aligned using Clustal X 2.0 [46] and adjusted manually using the similarity criterion [47]. The aligned sequences were analyzed using parsimony in PAUP*4.0b10 [48] with tree-bisection-reconnection branch-swapping. The ensemble retention index (RI) [49] was calculated for each of the 78 coding regions and 128 non-coding regions. The 10 coding and 10 non-coding regions with the highest percentages of parsimony-informative characters were then selected as candidates for phylogenetic markers.

#### Phylogenetic analysis

The 73 shared protein-coding genes from the plastid genomes in the seven Myrtales species and the three Geraniaceae outgroup species were aligned in Clustal X using the default settings, followed by manual adjustment to preserve the reading frames. The data matrix is posted as S1 Matrix. Three phylogenetic-inference methods were used to infer trees from these 73 concatenated genes. Parsimony analysis was implemented in PAUP* 4.0b10 [48], maximum likelihood (ML) in PHYML v 2.4.5 [50], and Bayesian inference (BI) in MrBayes 3.1.2 [51] using the settings from [22].

## Results and Discussion

### Sequencing, assembly and annotation

The whole plastid genome for *Lagerstroemia fauriei* was found to be 152,440 bp in length after combining the Sanger and Illumina sequence data. Through mapping the paired reads onto the finished genome, we verified our assembled length for the finished plastid genome with 1,473,293 (5% of the total reads) mapped reads across the whole genome with at least 951 reads per position. Based on this number of reads we consider the assembled genome to be of high-quality. Our annotated plastid genome of *L*. *fauriei* is available from GenBank (KT358807).

### Plastid genome features

In most land plants, the plastid genome is a single circular structure of 115–165 kb in length that consists of one large single-copy (LSC) region, one small single-copy (SSC) region, and a pair of inverted repeats (IRs). Although gene order and content are highly conserved in plastid genomes, they differ in the extent of gene duplication, size of intergenic spacers, presence or absence of introns, as well as the length and number of small repeats [52]. Such differences not only leave molecular patterns that allow for the inference of evolutionary history, but can also influence the molecular functioning of the cell as a whole (e.g., [20, 32]).

**Fig 1:**
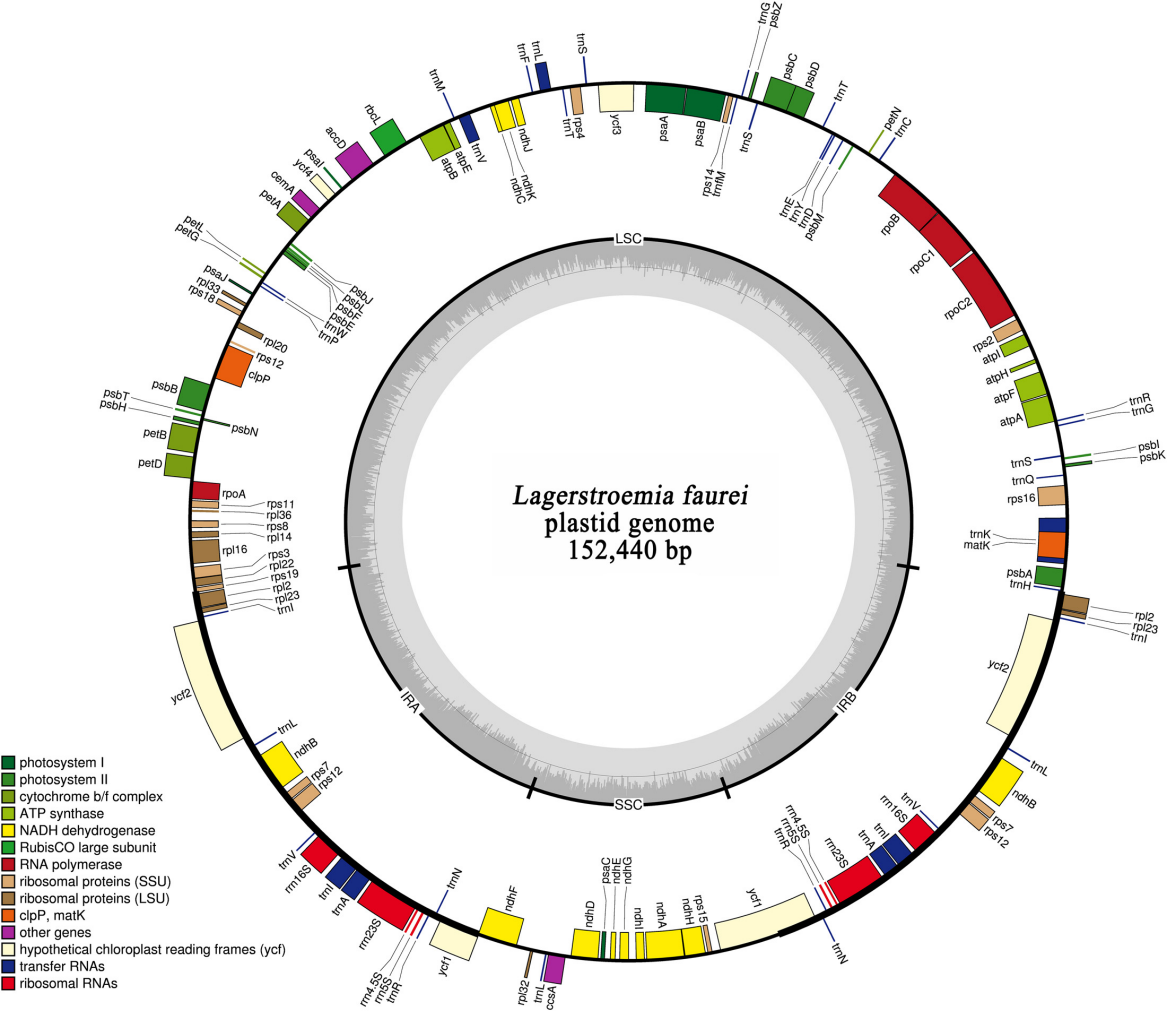
Map of the *L*. *fauriei* plastid genome. Genes shown outside the outer circle are transcribed clockwise and genes inside the outer circle are transcribed counterclockwise. Genes in different functional groups are color coded. The shaded area inside the inner circle indicates the GC content, with dark shading indicating percent CG.

The plastid genome of *L*. *fauriei* is composed of two single-copy regions separated by a pair of 25,793 bp IRs (Fig 1, Table 1), which account for 34% of the whole plastid genome. The LSC and SSC regions span 83,921 bp and 16,933 bp, respectively. The proportion of LSC and SSC length in the total plastid genome is 55% and 11%, respectively (Table 1). The *L*. *fauriei* plastid genome consists of protein coding genes, transfer RNA (tRNA), ribosomal RNA (rRNA), intronic and intergenic regions (Table 2). 81,412 bp (53%) of the whole *L*. *fauriei* plastid genome are non-coding DNA, 68,655 bp (45%) are protein-coding exons, 2,373 bp (2%) are tRNA, 4,517 bp (3%) are rRNA, 14,503 bp (10%) are intronic regions, and 62,570 bp (41%) are intergenic regions (Table 2).

**Table 1:** Comparison of plastid genome size among seven Myrtales species.

Region	*L*. *fauriei*	*O*. *argillicola*	*A*. *costata*	*C*. *eximia*	*E*. *aromaphloia*	*S*. *quadrifida*	*S*. *cumini*
LSC							
Length (bp)	83,923	88,511	88,768	88,522	88,925	88,247	89,081
GC content (%)	36	37	35	35	35	35	35
Length percentage (%)	55	54	55	55	56	55	56
SSC							
Length (bp)	16,933	19,000	18,772	18,672	18,468	18,544	18,508
GC Content (%)	31	35	30	31	31	31	31
Length Percentage (%)	11	12	12	12	12	12	12
IR							
Length (bp)	25,792	28,772	26,392	26,409	26,378	26,385	26,392
GC Content (%)	43	43	43	43	43	43	43
Length Percentage (%)	34	35	33	33	33	33	33
Total							
Length (bp)	152,440	165,055	160,326	160,012	160,149	159,561	160,373
GC Content (%)	38	39	37	37	37	37	37

**Table 2:** Comparison of coding and non-coding region size among seven Myrtales species.

Region	Species	*L*. *fauriei*	*O*. *argillicola*	*A*. *costata*	*C*. *eximia*	*E*. *aromaphloia*	*S*. *quadrifida*	*S*. *cumini*
Protein coding	Length (bp)	68,477	70,706	68,257	68,889	68,085	68,746	68,448
	GC content (%)	45	43	43	43	43	43	43
	Length percentage (%)	38	40	37	37	37	37	38
tRNA	length (bp)	2,373	2,303	3,184	2,199	2,270	2,387	2,310
	GC content (%)	54	53	49	53	53	52	53
	Length percentage (%)	2	1	2	1	1	2	1
rRNA	length (bp)	4,517	4,551	4,510	4,528	4,528	4,528	4,525
	GC content (%)	56	55	55	55	55	55	55
	Length percentage (%)	3	3	3	3	3	3	3
Intron	length (bp)	14,503	13,311	15,514	15,499	14,720	15,465	15,496
	GC content (%)	36	38	35	36	36	36	36
	Length percentage (%)	10	8	10	10	9	10	10
Intergenic	length (bp)	62,570	70,706	68,861	68,897	70,546	68,435	69,594
	GC content (%)	36	37	35	35	35	35	35
	Length percentage (%)	41	43	43	43	44	43	43

The plastid genome of *L*. *fauriei* contains 129 coding genes, including 84 protein-coding genes, 37 tRNA genes, and eight rRNA genes. Among the 129 genes, 4 rRNA genes, 7 tRNA genes and 6 coding genes are duplicated in the two IR regions (Fig 1; Table 3). Of the 112 unique genes, 82 are located in the LSC region (60 protein-coding genes, 22 tRNA genes), 13 in the SSC region (12 protein-coding genes, 1 tRNA gene), and 17 in both IR regions (6 coding genes, 4 rRNA genes, 7 tRNA genes). The following four genes span regional plastid boundaries: *ycf*1 spans the SSC and IR_B_ regions, *rps12* spans the LSC and two IR regions (5’ end exon was in LSC and two 3’end exons were duplicated in IR regions), *ndhF* spans the IR_A_ and SSC regions and *rps19* spans the LSC and IR_A_ region (Fig 1). In the whole plastid genome, 17 genes contain introns, including eight protein-coding genes with a single intron each (*atpF*, *ndhA*, *ndhB*, *petB*, *petD*, *rpl16*, *rpoC1*, *rps16*), five tRNA genes with a single intron each (*trnA*^*GUC*^, *trnG*^*UCC*^, *trnI*^*GAU*^, *trnK*^*UUU*^, *trnL*^*UAA*^, *trnV*^*UAC*^), and three protein coding genes with two introns each (*clpP*, *rps12* and *ycf3*). Among the 17 genes with introns, 13 genes are located in LSC, one in SSC, and three in both IRs (S2 Table). The *rps12* gene is a trans-spliced gene with a 5’ end exon in the LSC region and two duplicated 3’-end exons in IR regions. The 2,497 bp intron of *trnK*^*UUU*^ is the longest, but 1491 bp of it codes for the *matK* gene.

**Table 3:** List of genes in the *L*. *fauriei* plastid genome.

Gene category	Group of genes	Name of genes
Self-replication	Transfer RNA genes	*trnA-UGC*^a^^,^^b^ *trnC-GCA trnD-GUC trnE-UUC trnF-GAA trnfM-CAU trnG-UCC trnG-GCC trnH-GUG trnI-CAU*^b^ *trnI-GAU*^a^^,^^b^ *trnK-UUU*^a^ *trnL-CAA*^b^ *trnL-UAA*^a^ *trnL-UAG trnM-CAU trnN-GUU*^b^ *trnP-UGG trnQ-UUG trnR-ACG*^b^ *trnR-UCU trnS-GCU trnS-GGA trnS-UGA trnT-GGU trnT-UGU trnV-GAC*^b^ *trnV-UAC*^a^ *trnW-CCA trnY-GUA*
	Small subunit of ribosome	*rps2 rps3 rps4 rps7b rps8 rps11 rps12*^a^^,^^b^ rps14 rps15 rps16* *rps18 rps19*
	Ribosomal RNA genes	*rrn16*^b^ *rrn23*^b^ *rrn4*.*5*^b^ *rrn5*^b^
	Large subunit of ribosome	*rpl2*^b^ *rpl14 rpl16*^a^ *rpl20 rpl22 rpl23*^b^ *rpl32 rpl33 rpl36*
	DNA dependent RNA polymerase	*rpoA rpoB rpoC1*^a^ *rpoC2*
Photosynthesis	Subunits of photosystem I	*psaA psaB psaC psaI psaJ*
	Subunits of photosystem II	*psbA psbB psbC psbD psbE psbF psbHpsbI psbJ psbK psbL psbM psbN psbT psbZ*
	Subunits of cytochrome	*petA petB*^a^ *petD*^a^ *petG petL petN*
	Subunits of ATP synthase	*atpA atpB atpE atpF*^a^ *atpH atpI*
	ATP-dependent protease subunit p gene	*clpP*^a^
	Large subunit of Rubisco	*rbcL*
	Subunits of NADH dehydrogenase	*ndhA*^a^ *ndhB*^a^^,^^b^ *ndhC ndhD ndhE ndhF ndhG ndhH ndhI ndhJ ndhK*
Other genes	Maturase	*matK*
	Envelop membrane protein	*cemA*
	Subunit of acetyl-CoA-carboxylase	*accD*
	c-type cytochrome synthesis gene	*ccsA*
Genes of unknown function	Conserved open reading frames	*ycf1 ycf2*^b^ *ycf3*^a^ *ycf4*

^a:^ Genes containing introns;

^b:^ Duplicated gene (Genes present in the IR regions).

### Comparison of the plastid genomes with six other Myrtales

We compared the plastid genome of *L*. *fauriei* (Lythraceae) to six other species in the Myrtales with dot-plot analysis. The plastid genomes in these species possess identical gene order with the exception of *O*. *argillicola*, which contains a large inversion of about 56 kb in the LSC region (S1 Fig) [53,54]. These results further verified the conserved feature of the plant plastid genome and partial lineage-specific variation [19]. The seven plastid genomes vary in length from 152,440 to 165,055 bp. From the comparative results (Table 1), the plastid genome of *O*. *argillicola* is the longest of the seven species, which is explained partly by expansion of intergenic regions in the SSC and IR regions. However, the plastome of *L*. *fauriei* is the shortest because of reduction of intergenic regions, which only occupy 41% of the genome (Table 2). These comparisons demonstrate that the dynamic variation of the intergenic regions is the main cause of length differences between plastid genomes [19, 22].

The GC content of the plastid genome is stable across most land plants [19]. The GC content of the entire *L*. *fauriei* plastid genome is 38%, with 36% GC content in the LSC region, 31% in the SSC region and 43% in the IR regions. These percentages are generally similar to other plastid genomes [55]. The overall GC contents in seven Myrtales plastid genomes ranged from 37% to 39%, with *O*. *argillicola* having the highest GC content and *A*. *costata* having the lowest (Table 1). The GC content of protein-coding regions in the seven Myrtales species range from 37% to 40%, of which *O*. *argillicola* has the highest and *C*. *eximia* has the lowest (Table 1).

**Fig 2:**
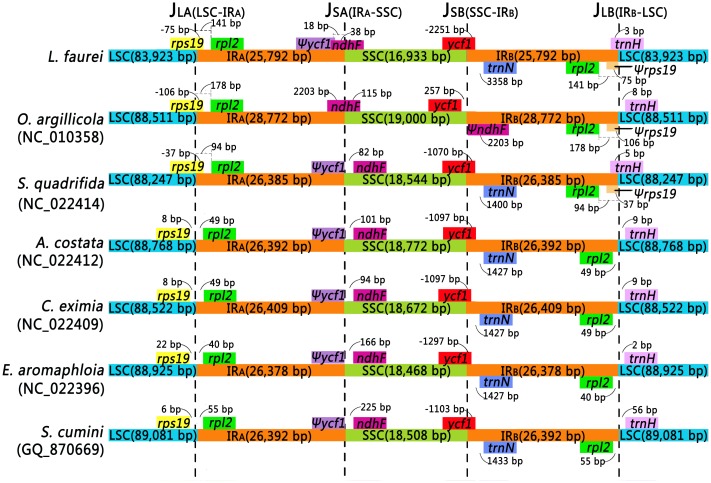
Comparison of junctions between the LSC, SSC, and two IR regions among seven Myrtales species. ψ means pseudogene; distance in the figure is not to scale.

From these cross-species comparisons, we verified that the Myrtales plastid genomes are highly conserved in genome content, gene order and overall genomic structure relative to *L*. *fauriei*. They have similar gene orders at the IR-SSC and IR-LSC borders, with the exception of *ψycf1* (pseudogene *ycf1*), which is absent from the border of IR_A_ and SSC in *O*. *argillicola*. Instead *O*. *argillicola* has a ψ*ndhF* (pseudogene *ndhF*) on the border of SSC and IR_B_ (Fig 2).

### Expansion and contraction of four junction regions

The typical quadripartite structure of plastomes includes two single-copy regions and two inverted repeat regions, though length of the IRs differ between plant species because of contraction and expansion in these regions [19]. We examined the four junctions (J_LA_, J_LB_, J_SA_, and J_SB_) across the seven Myrtales species to assess the junction variation between the IRs and single-copy regions following Wang [40] and Wu [22].

The length of the IRs ranged from 25,792 to 28,772 bp, and the positions of all four IR boundaries (J_LA_, J_LB_, J_SA_, and J_SB_) varied (Fig 2) [56]. The LSC/IR_A_ junctions in plastid genomes of *L*. *fauriei*, *O*. *argillicola*, and *S*. *quadrifida* were located in the coding region of *rps19*, which extended into the IR_B_ region 75 bp, 106 bp, and 37 bp, respectively. In the other four species the LSC includes an intact *rps19* gene together with 8 bp (*A*. *costata*, *C*. *eximia*), 22 bp (*E*. *aromaphloia*), or 6 bp (*S*. *cumini*) of non-coding region beyond the LSC/IR_A_ border. The IR_B_/LSC border in these four species is located in the intergenic spacer between *rpl2* and *trnH*. The *trnH* gene of *S*. *cumini* is 56 bp away from the IR_B_/SSC border, whereas in *L*. *fauriei* and *S*. *quadrifida* the *trnH* gene extends into the IR_B_ by 3 bp and 5 bp respectively. In the other four species the *trnH* gene is 2–9 bp away from the IR_B_/SSC border.

In *O*. *argillicola*, the *ycf1* gene does not extend into the IR_B_ region at the border of SSC/IR_A_. Rather, in contrast to the other six species wherein *ycf1* extends across the border, *ycf1* in *O*. *argillicola* is separated by 257 bp. Hence the SSC/IR_B_ junction resulted in the duplication of the 3’ end region of *ycf1* in these six species, and consequently a pseudogene with variable length at the IR_A_/SSC border (Fig 2) [49].

Variable gene composition was found at the IR_A_/SSC border. In *O*. *argillicola* the *ψycf1* gene is absent, and instead the IR_A_/SSC border was positioned in the *ndhF* gene, which had 115 bp in the SSC region and 2,203 bp in the IR_A_ region. Similarly, *ndhF* extends 38 bp into the IR_A_ region in *L*. *fauriei*, which also has 20 bp overlap with *ψycf1*. The entire *ndhF* gene is located in the SSC region in the other five species and is separated by 82–225 bp from the IR_A_/SSC border. The IR/LSC border region has been used extensively for phylogenetic studies in *Eucalyptus* [36,57] and given the variation we observed, this region could be similarly useful for resolving the relationships between *L*. *fauriei* and its relatives.

### Loss of the *rpl2* intron from *Lagerstroemia* and Lythraceae

**Fig 3:**
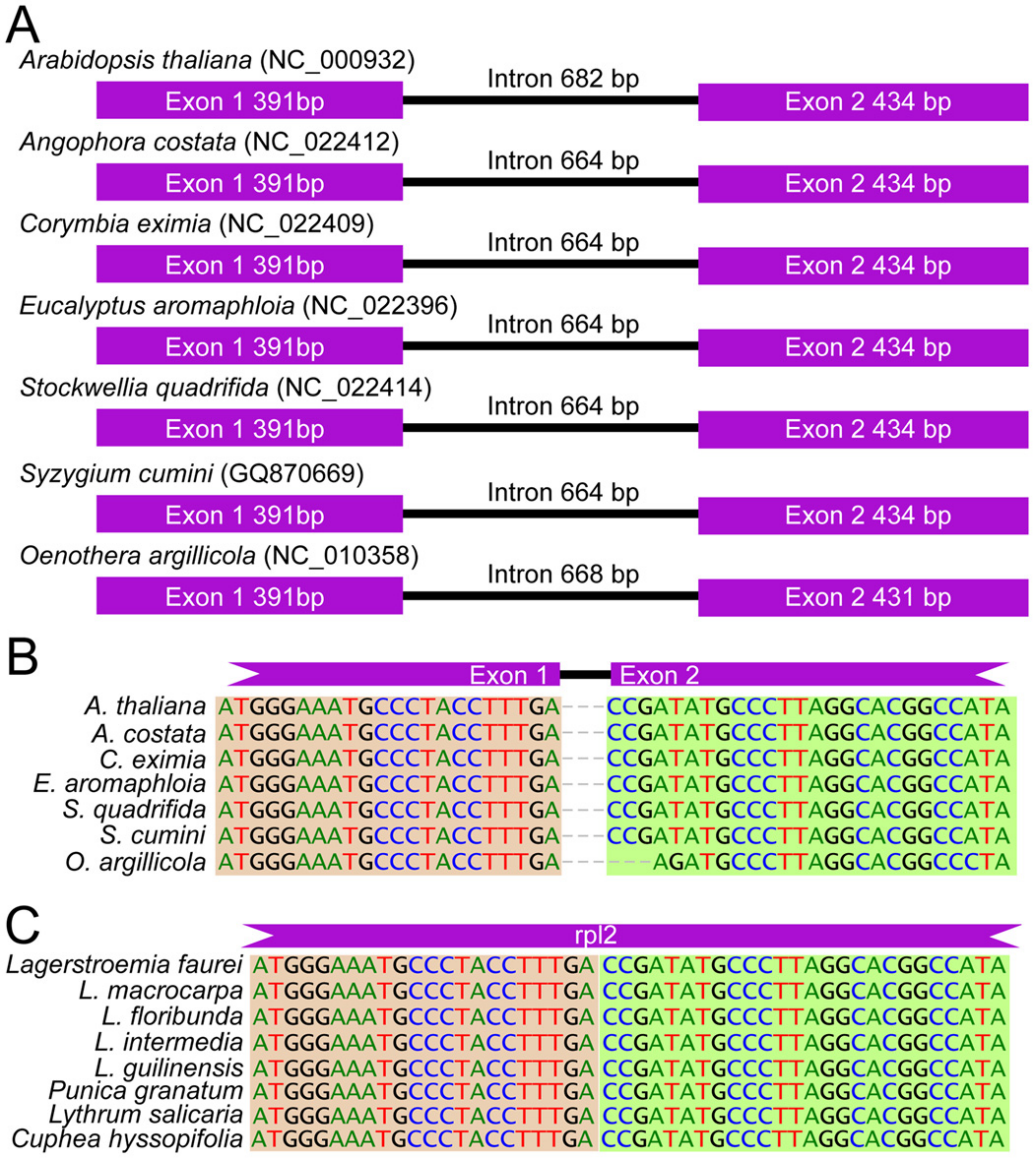
The structure and sequence variation for *rpl2* gene with and without intron. **(A)** The structural components of *rpl2* gene from *Arabidopsis thaliana* (NC_000932) and the other six Myrtales species. **(B)** The boundary sequences of the two exons: the dashed lines represent the intron sequences; the sequence from the maple shade is from first exon and from the green shade is from the second intron. **(C)** Exons borders of *rpl2* sequences from genus *Lagerstroemia* and other species from Lythraceae: the maple and green shades mean the sequence from exon 1 and 2.

The distribution and number of introns in the *L*. *fauriei* plastid genome are similar to other Myrtales plastid genomes (S2 Table), with the exception of the intron of *rpl2*. The structure and the length of the intron for *rpl2* is conserved across all other Myrtales and also present in the more distant *Arabidopsis thaliana* (NC_000932; Fig 3A). The length of this intron is approximately 660 bp in the other sampled six Myrtales species and the two exons are also highly conserved. To verify the loss of the *rpl2* intron in the whole *Lagerstroemia* or even broadly within Lythraceae as a whole, we designed a pair of primers in the flanking exons to amplify and sequence the region spanning the intron among different species. From the *rpl2* gene alignment, the intron was absent among all 14 Lythraceae species sampled (S2 and S3 Figs), but the intron was present in *Oenothera albicaulus* (Fig 3B; from the arrow of S2A Fig). From the PCR amplification test (S3 Fig), the *rpl2* amplicon is about 750 bp in 14 samples species of Lythraceae, whereas in the amplicons from the outgroups *O*. *albicalus* and *Catha edulis* were about 1,400 bp (S3 Fig). These results indicate that the intron was lost after the divergence of the Lythraceae from the Onagraceae (S2B and S3 Figs) but prior to the divergence of the four Lythraceae genera sampled.

Plastid introns have been lost numerous times in other species, such as those reported from the legume tribe Desmodieae [58,59], and have been documented in both monocots and dicots [60]. Specifically, *rpl2* intron loss has been reported from five other lineages of dicotyledons: Saxifragaceae, Convolvulaceae, Menyanthaceae, two genera of Geraniaceae, and one genus of Droseraceae [59]. The discovery of this intron loss indicates a structural difference between Lythraceae and the six other Myrtales families sampled. And we could confirm that many times instances of independent intron loss have happened in the history of plastid genome evolution. Two different theories had been proposed to explain loss of the *rpl2* intron [61,62]. First, through the homologous recombination, the full *rpl2* transcript (cDNA) could replace the *rpl2* gene by the reverse-transcriptase mediated mechanism to precisely delete the entire intron. Alternatively, *rpl2* intron loss could be caused by unknown processes involving intron removal by DNA-level deletion or gene conversion between an intron-containing gene and its spliced transcript. In near future, by combining the density samplings within Lythraceae and Onagraceae, and by employing the data from RNA and DNA could answer this intron loss history around this family.

### Long repetitive sequences

Long repetitive sequences have an important role in structural variation in plastid genomes via recombination and rearrangement [63]. Tandem repeats (≥15 bp), and forward and palindromic repeats (≥30 bp) were compared across the seven Myrtales species (S4B Fig). Most of these repeats are located in intergenic spacers, except for some that are distributed in the shared coding regions of *ycf2* and *psaB*. *L*. *faurei* has the fewest (22) repeats, which is consistent with the small genome size of *L*. *fauriei* compared with the six other Myrtales species sampled (S4B Fig).

Repeated sequences have been demonstrated to affect genome length [64]. Our data are consistent with these findings given that the length and number of repeat in *O*. *argillicola* and *L*. *fauriei* (S4 Fig) are correlated with their genome size. Forward-repeat sequences are often associated with transposons [65], which can proliferate during episodes of cellular stress [66, 67]. The origins and proliferation of large tandem repeats are not as well understood as interspersed repetitive sequences [68]. Forward repeats can cause genomic reconfiguration, and therefore have potential to be useful markers in phylogenetic studies.

### Plastid SSRs

Simple sequence repeats (SSRs) in the plastid genome can be highly variable at the intraspecific level, and therefore valuable markers for population-genetic studies [56]. We identified 204 SSRs in the plastid genome of *L*. *fauriei*, of which 132 are located in non-coding regions and 72 in coding regions. These SSRs include 115 mononucleotide SSRs (homopolymers; 56%), 35 dinucleotide SSRs (17%), 46 trinucleotide SSRs (23%), seven tetranucleotide (3%), and one pentanucleotide SSR (1%). Of the 204 SSRs, 143 are in the LSC region, 35 in SSC, and 26 in IR_A_ region accounting for 70%, 17%, and 13% of the total SSRs, respectively. Among the 115 homopolymer SSRs, 113 (98%) are the A/T type with a repeat number from 8 to 14. Among the coding regions, *ycf2* was found to possess 13 SSRs, followed by *ycf1* with eight SSRs. This result is consistent with previous studies which found that these genes are highly variable in other species [67, 68, 69]. From this result *ycf1*and *ycf2* are potential candidates for species-level DNA barcoding[70].

Among the seven Myrtales species sampled, *L*.*faurei* has the fewest SSRs (S4C Fig). The total length of SSRs in these species does not have a strong overall correlation to genome size. However *L*. *fauriei* has the shortest chloroplast genome and had the smallest contribution from SSRs. Thus, reduction in the size and presence of SSR’s may contribute somewhat to the short chloroplast genome of *L*. *fauriei* [71].

### Highly informative regions and potential markers for phylogenetic analysis

Identifying highly variable gene regions provides an important resource for phylogenetic analyses and DNA barcoding [72]. Regions such as *atpB*, *atpB-rbcL*, *matK*, *ndhF*, *rbcL*, *rpl16*, *rps4-trnS*, *rps16*, *trnH-psbA*, *trnL-F*, and *trnS-G* have been extensively employed for phylogenetic reconstructions [73–75] and barcoding applications [76,77]. Using complete plastid genomes, we identified additional informative loci for use within the Myrtales, including *Lagerstroemia*.

We aligned all coding and non-coding regions ≥ 200 bp separately to identify regions with the highest percentage of parsimony-informative sites, and the highest ensemble retention index, among the seven Myrtales species sampled (Table 4, S3 Table). Among the coding regions, *rpoA* and *matK* have the highest percentage of parsimony-informative characters (7% and 6%, respectively). Among non-coding regions, *trnR*^*UCU*^*-atpA* and *trnK*^*UUU*^*-rps16* have the highest percentages (20% and 14%, respectively). These non-coding regions should be particularly informative for DNA barcoding and species-level phylogenetic analyses within the Myrtales given the high percentage of variable sites (S3 Table). In order to better understand the variation from the longer genes (>1500 bp) and make them usable in practical applications, we employed the sliding-window method (S4 Table). By applying this method, we identified the most variable regions within each gene that would be valuable as molecular makers in phylogeny or for marker-assisted breeding analysis. For example, the most variable region of *ycf1*, which is over 7000 bp in length, is located from 5 to 6 kb downstream from the start.

**Table 4:** Top ten coding regions ordered with respect to their potential phylogenetic signal.

No.	Region	Length (bp) ^a^	Aligned length (bp) ^b^	Conserved sites	Pars. Inf. ^c^	Pars. Inf.% ^d^	RI^e^
1	*rpoA*	1002	1101	989	77	6.99	0.96
2	*matK*	1500	1593	1295	98	6.15	0.92
3	*rps15*	273	297	253	15	5.05	0.86
4	*rpl22*	471	486	396	24	4.94	0.82
5	*rpl32*	174	306	271	15	4.90	0.77
6	*ccsA*	960	966	827	47	4.87	0.80
7	*ndhF*	2244	2349	1967	113	4.81	0.82
8	*ycf1*	5613	7356	5102	350	4.76	0.70
9	*ndhG*	531	555	496	24	4.32	0.88
10	*petL*	96	96	91	4	4.17	0.75

^a:^ Length: refers to sequence length in *L*.*fauriei*;

^b:^ Aligned length: refers to the alignment of seven Myrtales species considered in the comparative analysis (see Materials and Methods);

^c:^ Number of parsimony informative sites;

^d:^ Percentage of parsimony informative sites;

^e:^ RI-Ensemble retention index.

Shaw [25,78] evaluated the phylogenetic utility of noncoding plastid regions and found that those that are most commonly used for phylogenetic analyses (e.g., *trnL* intron, *trnL*-*trnF* spacer) are among the least variable. Thus, our identification of ten more variable noncoding regions provides a valuable resource for future phylogenetic studies within Myrtales, including our focal genus, *Lagerstroemia*.

### Phylogenetic analysis

**Fig 4:**
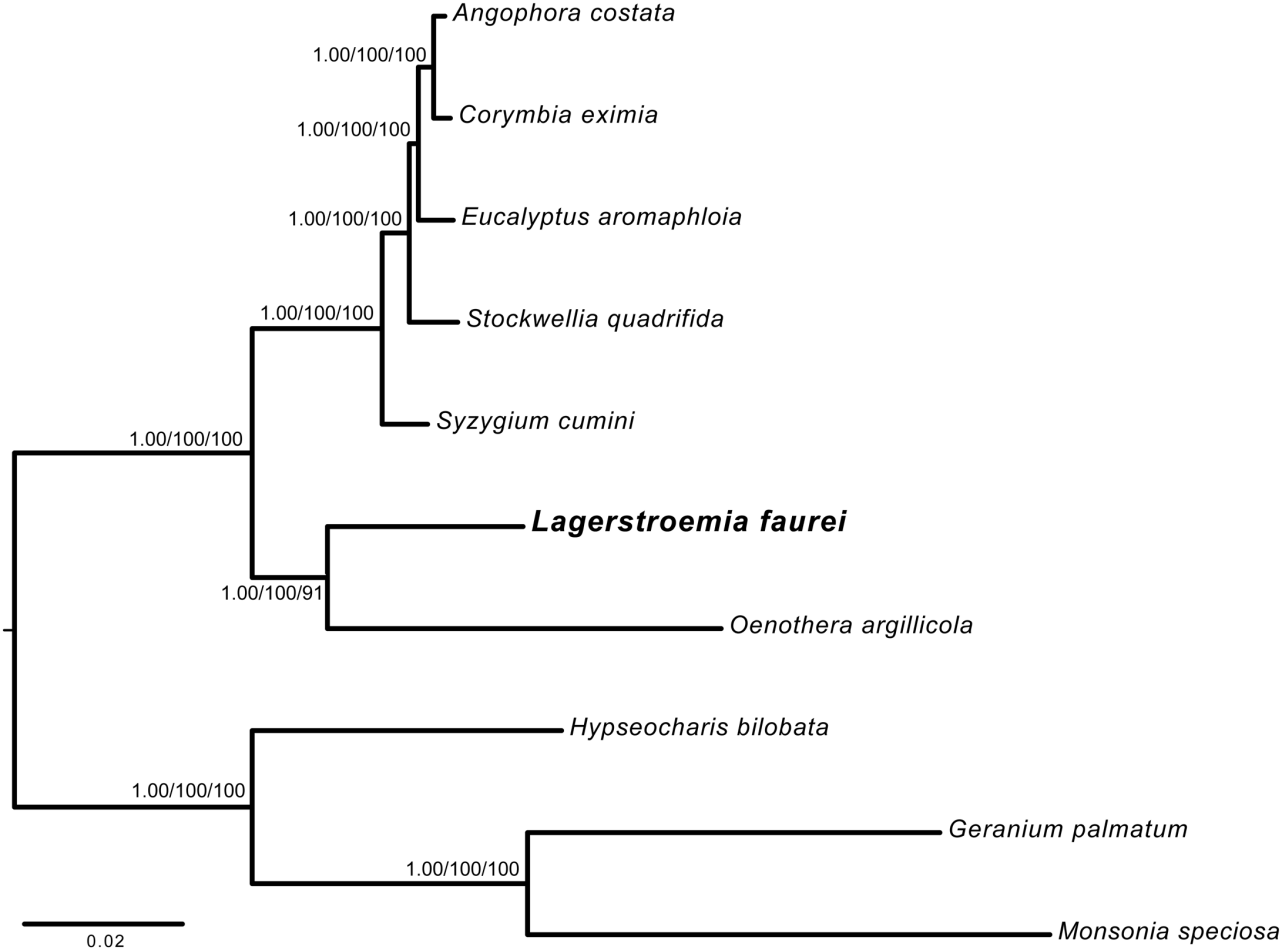
Phylogenetic tree inferred by Bayesian inference, maximum likelihood, and parsimony using 73 shared protein-coding genes among 10 plastid genomes (1 Lythraceae, 1 Onagraceae, 5 Myrtaceae, 3 Geraniaceae). Numbers above nodes indicate posterior probability followed by bootstrap values.

Phylogenetic analysis using plastid sequences have resolved numerous lineages within the angiosperms [79,80]. Furthermore, *atpF-atpH*, *matK*, *psbK-psbI*, *rbcL* and *trnH-psbA* have been used successfully as species-level barcodes [76,81,82]. Phylogenetic relationships within Lythraceae have been inferred using morphology and DNA sequences from the *rbcL* gene, the *trnL-F* region, and the *psaA-ycf3* intergenic spacer from the plastid genome, together with ITS from the nuclear genome [1,17]. Our phylogenetic analyses included seven Myrtales species together with three outgroups from Geraniaceae. These analyses all corroborated the sister relationship between Lythraceae and Onagraceae based on 73 shared protein-coding genes (Fig 4). From the branch-length differences between the two main Myrtales clades, we infer that both Lythraceae and Onagraceae have undergone a more rapid rate of nucleotide substitution than their Myrtaceae sister group. This more rapid nucleotide-substitution rate was also accompanied by more structural differences in the Onagraceae and Lythraceae.

## Supporting Information

S1 Fig Dot-plots comparing the *L*. *fauriei* plastid genome to those of six other Myrtales species. (TIF)

S2 Fig The Sanger sequence verification of the *rpl2* gene from species with and without the intron. (A) The boundary sequences of two exons: the dash lines represents the elliptical intron sequences; the sequence from the maple shade is from first exon and from the green shade is from the second intron. The Sanger sequencing chromatograms with first exon and intron regions was from *O*. *albicaulus*. (B) The joints of two exons of *rpl2* sequences from genus *Lagerstroemia* and other species from Lythraceae: the maple and green shades mean the sequence from exon 1 and 2. The Sanger sequencing chromatograms from five species from Lythraceae show the loss of intron.
(TIF)

S3 Fig PCR products indicating *rpl2* intron absence in *Lagerstroemia*. MA = *L*. *macrocarpa*, FL = *L*. *floribunda*, INT = *L*. *intermedia*, GU = *L*. *guilinensis*, FA = *L*. *fauriei*, VE = *L*. *venusa*, CAU = *L*. *caudata*, LI = *L*. *limii*, SUB = *L*. *subcostata*, IND = *L*. *indica*, PA = *L*. *parvifolia*, PU = *Punica granatum*, LY = *Lythrum salicaria*, CU = *Cuphea hyssopifolia*, OEN = *Oenothera albicaulus*, CATHA = *Catha edulis*, C = negative control.
(TIF)

S4 Fig Lengths of plastid genomes and repetitive regions. A. Plastid genome size comparison among seven Myrtales species (1 = *Lagerstroemia fauriei*, 2 = *Oenothera argillicola*, 3 = *Angophora costata*, 4 = *Corymbia eximia*, 5 = *Eucalyptus aromaphloia*, 6 = *Stockwellia quadrifida*, 7 = *Syzygium cumini*, with species listed according to their distance). B. All repeat sequences, tandem repeats (≥15 bp), and forward and palindromic repeats (≥30 bp) for each of seven Myrtales species. Bars indicate total length of each type of repeat. C. Total length contribution from SSRs for each of seven Myrtales species, separated by motif type.
(TIF)

S1 Matrix Supplementary matrix: The full alignment of 73 protein-coding genes from 10 used species (NEXUS format). (NEX)

S1 Table Primers used for gap closure in *L*. *fauriei*. (DOCX)

S2 Table Lengths of exons and introns in intron-containing genes from the plastid genome of *L*. *fauriei*. (DOCX)

S3 Table Ten highest sites of non-coding regions with respect to their potential phylogenetic signal. (DOCX)

S4 Table Divided genes (longer than 1.5kb) into short regions and their parsimony-informative distribution. (DOCX)
