# Supplementary figures and images for: The Complete Plastid Genome of Lagerstroemia fauriei and Loss of rpl2 Intron from Lagerstroemia (Lythraceae)
Source: PLoS One. 2016 Mar 7;11(3):e0150752. doi: 10.1371/journal.pone.0150752 (PMC4780714; doi:10.1371/journal.pone.0150752)

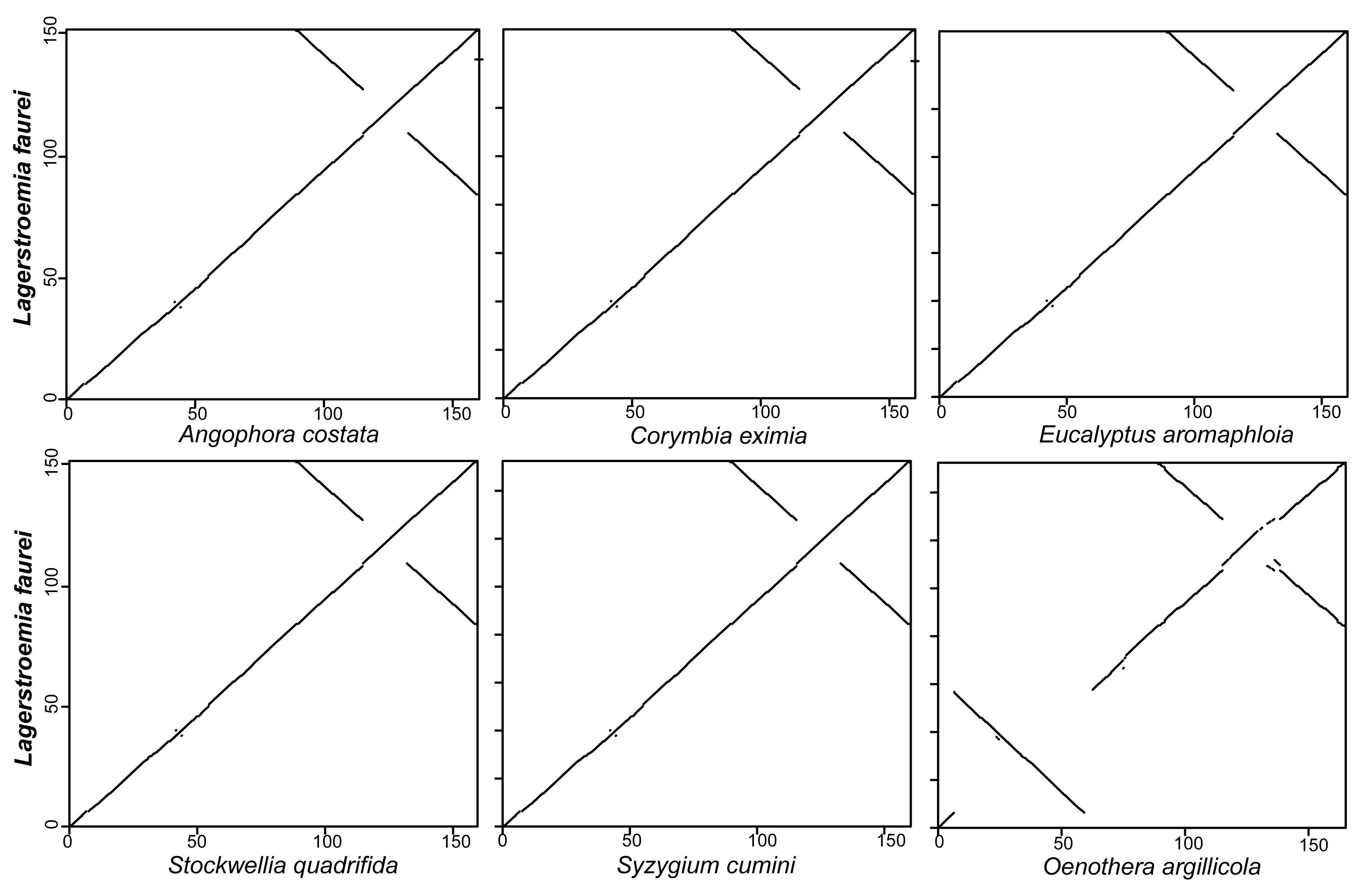

Supplement: S1 Fig — (TIF) [file pone.0150752.s001.tif]

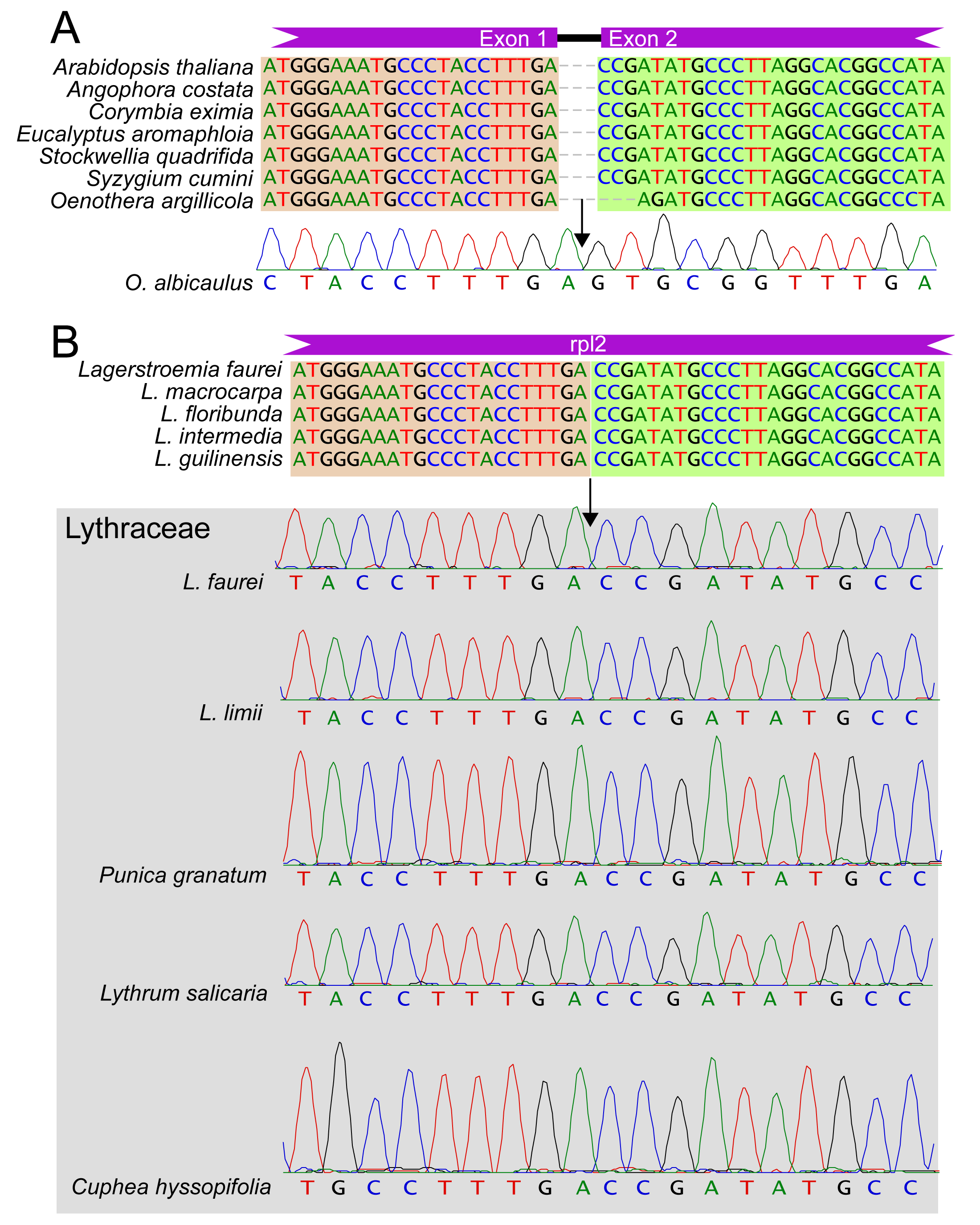

Supplement: S2 Fig — (A) The boundary sequences of two exons: the dash lines represents the elliptical intron sequences; the sequence from the maple shade is from first exon and from the green shade is from the second intron. The Sanger sequencing chromatograms with first exon and intron regions was from O. albicaulus. (B) The joints of two exons of rpl2 sequences from genus Lagerstroemia and other species from Lythraceae: the maple and green shades mean the sequence from exon 1 and 2. The Sanger sequencing chromatograms from five species from Lythraceae show the loss of intron. (TIF) [file pone.0150752.s002.tif]

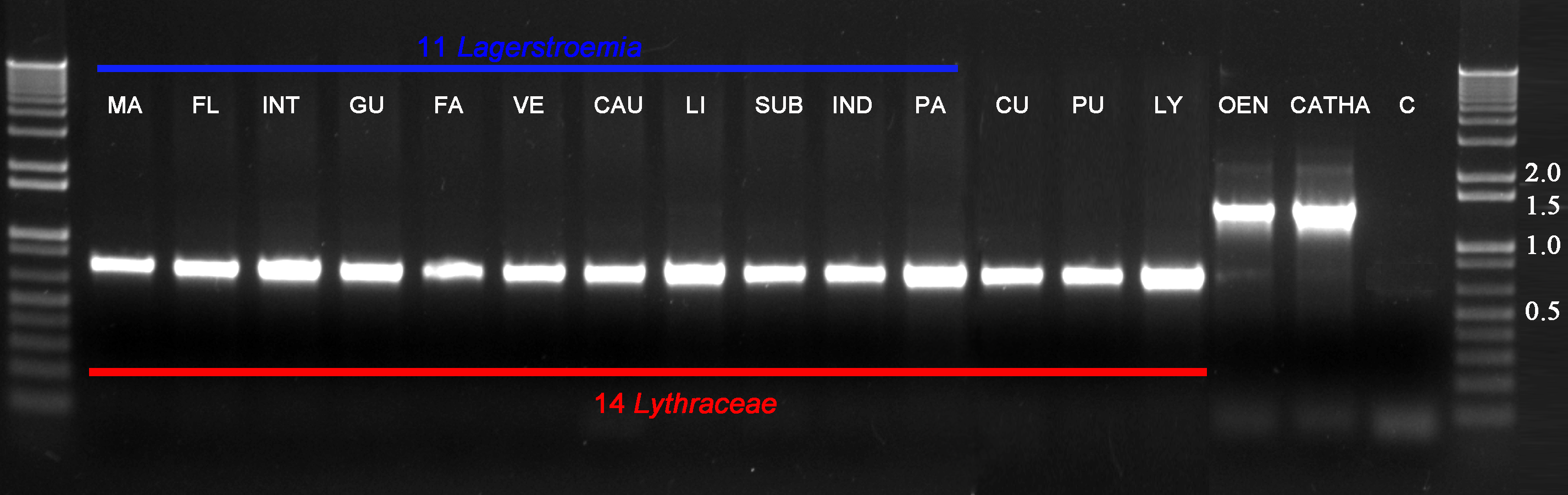

Supplement: S3 Fig — MA = L. macrocarpa, FL = L. floribunda, INT = L. intermedia, GU = L. guilinensis, FA = L. fauriei, VE = L. venusa, CAU = L. caudata, LI = L. limii, SUB = L. subcostata, IND = L. indica, PA = L. parvifolia, PU = Punica granatum, LY = Lythrum salicaria, CU = Cuphea hyssopifolia, OEN = Oenothera albicaulus, CATHA = Catha edulis, C = negative control. (TIF) [file pone.0150752.s003.tif]

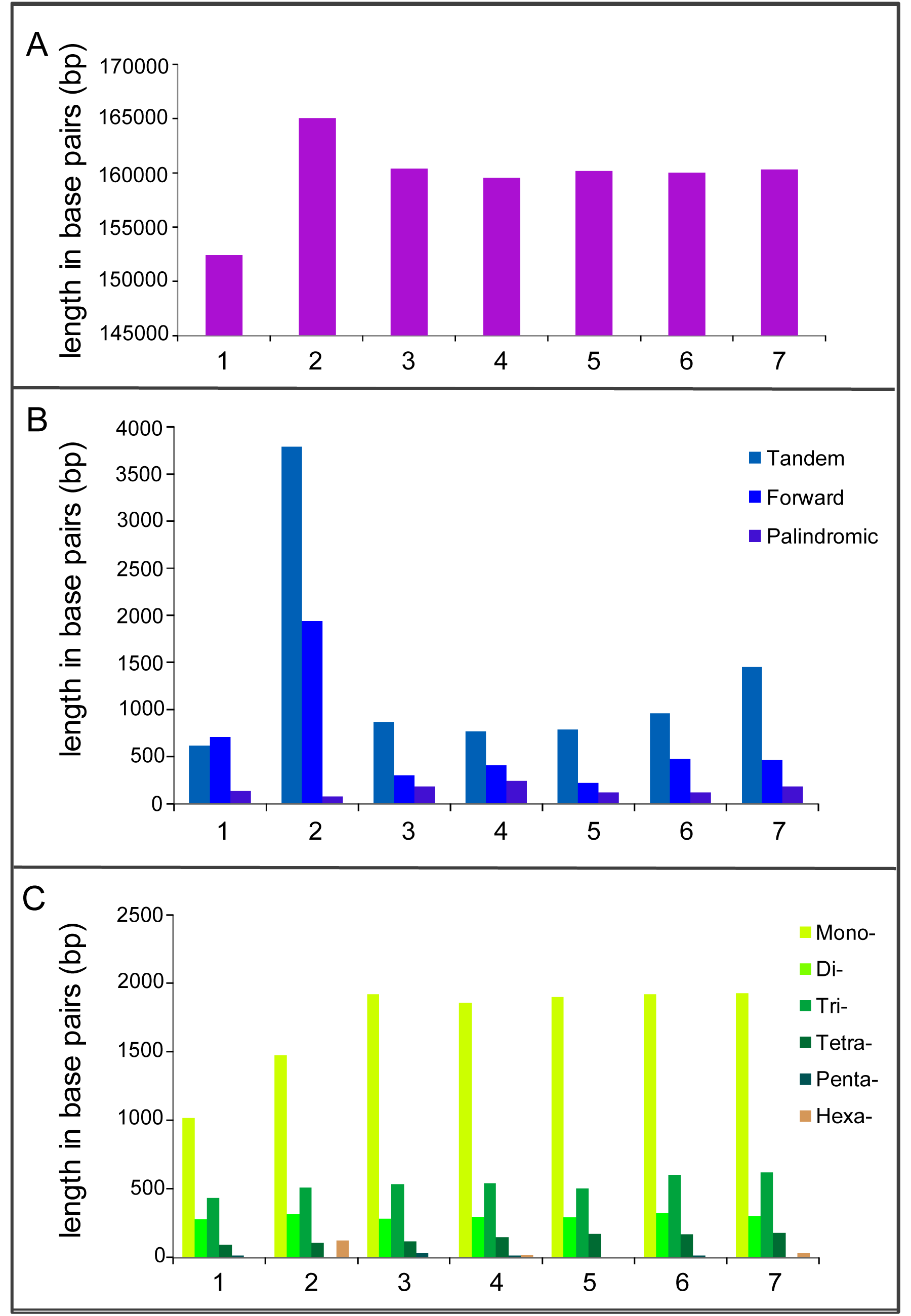

Supplement: S4 Fig — A. Plastid genome size comparison among seven Myrtales species (1 = Lagerstroemia fauriei, 2 = Oenothera argillicola, 3 = Angophora costata, 4 = Corymbia eximia, 5 = Eucalyptus aromaphloia, 6 = Stockwellia quadrifida, 7 = Syzygium cumini, with species listed according to their distance). B. All repeat sequences, tandem repeats (≥15 bp), and forward and palindromic repeats (≥30 bp) for each of seven Myrtales species. Bars indicate total length of each type of repeat. C. Total length contribution from SSRs for each of seven Myrtales species, separated by motif type. (TIF) [file pone.0150752.s004.tif]
