# Supplementary material for: The Complete Plastid Genome of Lagerstroemia fauriei and Loss of rpl2 Intron from Lagerstroemia (Lythraceae)
Source: PLoS One. 2016 Mar 7;11(3):e0150752. doi: 10.1371/journal.pone.0150752 (PMC4780714; doi:10.1371/journal.pone.0150752)
Supplement: S1 Table — (DOCX) [file pone.0150752.s006.docx]

**S1 Table. Primers used for gap closing, junction and sequencing verification in *L. fauriei***

| Number | Primers | Sequence | Information |
| --- | --- | --- | --- |
| 1 | tRNA-Asn_F36 | TTAACAGCCGACCGCTCTAC | Gap Closing |
|  | SSC_1423bp_R604 | CCCCATGAATCGGACAATAC |  |
| 2 | SSC_1423bp_F1201 | CGGCTCGATAAGAACCCATA | Gap Closing |
|  | SSC_1352bp_R163 | CAGCGTGTCTACCGATTTCA |  |
| 3 | SSC_1352bp_F1064 | TGGGACCCAAAAGAAACTTG | Gap Closing |
|  | SSC_4493bp_R218 | TCCCTGATTCGGTCTCTGAC |  |
| 4 | SSC_4493bp_F257 | AACAAGAACCAATCCCAACG | Gap Closing |
|  | SSC_6252bp_R352 | GATCTCCCCGTTGTTGATTG |  |
| 5 | SSC_6252bp_F395 | TTTCTTGGATGGCTGGATTC | Gap Closing |
|  | IR_1290bp_R549 | AATCCCCTTCCATTCTTTGG |  |
| 6 | IR_30407_F238 | GCCGAGGACTCTACCATTGA | Gap Closing |
|  | LSC_IR_93715bp_R | CCAATGAGCCCTTTATCGAA |  |
| 7 | ndhF_F229 | AAAACAGCAATGCTTTGGAA | Gap Closing |
|  | ccsA_R190 | CCGAATAAATCCAACGAGTG |  |
| 8 | p1-p2-F3573_860bp | CAATCGCTCTTTTGATTTTG | Gap Closing |
|  | p1-p2-R4436_860bp | TGCTAATCCAGCACAAGTCT |  |
| 9 | p1-p2-F4418_650bp | GACTTGTGCTGGATTAGCAT | Gap Closing |
|  | p1-p2-R5047_650bp | TATCCCAATGAGCCCTTTAT |  |
| 10 | p1-p2-F29240_1.1Kb | TGATCCAACCCCTTTATTTT | Gap Closing |
|  | p1-p2-R30251_1.1Kb | CATCGTCTAGTGGTTCAGGA |  |
| 11 | p1_F67003_880bp | GGGCTTTAGCAGGTCTATTG | Gap Closing |
|  | P1_R67883_880bp | AATATGCCCCTTTTCATGTT |  |
| 12 | P2_F111644_770bp | GAACAAAAATTCCAACGGTA | Gap Closing |
|  | P2_R112413_770bp | CAAGCAAGTTATGAGCCATC |  |
| 13 | P2_F114556_600bp | TTGGGCATTTATTACTTGGA | Gap Closing |
|  | P2_R115159_600bp | CATGATACGCCAAATGTTCT |  |
| 14 | 7210-7350_F34 | CTGTCTTAATTCCGCCCTTT | Sequence Correction |
|  | 7210-7350_R1351 | GCCCTATCTGTTCTGTCCAA |  |
| 15 | 29670-30070_F170 | CGCTATCGCCTTTCTAATACA | Sequence Correction |
|  | 29670-30070_R1059 | TTGCTAACAACCCAATTTCC |  |
| 16 | 51230-51440-F32 | TGTCAAGCCATCCATAACTG | Sequence Correction |
|  | 51230-51440-R1184 | AAGGTCTACGGTTCGAGTCC |  |
| 17 | 76130-76360_F239 | TGGTAGTTCGATCGTGGAAT | Sequence Correction |
|  | 76130-76360_R1387 | GATCGGATTAACCAACCAAA |  |
| 18 | 113120-113310-F134 | AAGAACAAAAATTCCAACGGTA | Sequence Correction |
|  | 113120-113310-R1261 | GGAACTGCCATTAAAAATAAAA |  |
| 19 | 117660-117930-F322 | CCATCTATTCCCAATCTCCA | Sequence Correction |
|  | 117660-117930-R1392 | TTGTTTCGGCAATTTATCGT |  |
| 20 | F3828 | TATTCATTCGACCCAACTCG | Sequence Correction |
|  | R5130 | AGCCGTACGAGGAGAAAACT |  |
