# Supplementary material for: The Complete Plastid Genome of Lagerstroemia fauriei and Loss of rpl2 Intron from Lagerstroemia (Lythraceae)
Source: PLoS One. 2016 Mar 7;11(3):e0150752. doi: 10.1371/journal.pone.0150752 (PMC4780714; doi:10.1371/journal.pone.0150752)
Supplement: S2 Table — (DOCX) [file pone.0150752.s007.docx]

**S2 Table. Lengths of exons and introns in intron-containing genes from the plastid genome of *L. fauriei***

| **Gene Name** | **Location** | **Exon I (bp)** | **Intron I (bp)** | **Exon II (bp)** | **Intron II (bp)** | **Exon III (bp)** |
| --- | --- | --- | --- | --- | --- | --- |
| *rps16* | LSC | 40 | 839 | 227 |  |  |
| *rpoC1* | LSC | 453 | 741 | 1608 |  |  |
| *atpF* | LSC | 145 | 776 | 410 |  |  |
| *petB* | LSC | 6 | 767 | 642 |  |  |
| *petD* | LSC | 8 | 727 | 475 |  |  |
| *ndhB* | IR | 777 | 686 | 756 |  |  |
| *ndhA* | SSC | 552 | 1043 | 540 |  |  |
| *rpl16* | LSC | 9 | 906 | 399 |  |  |
| *rps12^a^* | LSC | 114 |  | 231 | 548 | 27 |
| *ycf3* | LSC | 126 | 748 | 228 | 754 | 153 |
| *clpP* | LSC | 71 | 833 | 292 | 593 | 228 |
| *trnK-UUU* | LSC | 37 | 2497 | 35 |  |  |
| *trnL-UAA* | LSC | 37 | 529 | 50 |  |  |
| *trnV-UAC* | LSC | 38 | 599 | 37 |  |  |
| *trnI-GAU* | IR | 42 | 937 | 35 |  |  |
| *trnA-UGC* | IR | 38 | 816 | 35 |  |  |
| *trnG-UCC* | LSC | 23 | 735 | 47 |  |  |

a: *rps12* is a trans-spliced gene with exon I located in the LSC region and the exon II and exon III located in IR region.
