# Supplementary material for: The Complete Plastid Genome of Lagerstroemia fauriei and Loss of rpl2 Intron from Lagerstroemia (Lythraceae)
Source: PLoS One. 2016 Mar 7;11(3):e0150752. doi: 10.1371/journal.pone.0150752 (PMC4780714; doi:10.1371/journal.pone.0150752)
Supplement: S3 Table — (DOCX) [file pone.0150752.s008.docx]

**S3 Table. Ten highest sites of non-coding regions with respect to their potential phylogenetic signal**

| **No.** | **Region** | **Length (bp) ^a^** | **Aligned length (bp) ^b^** | **Conserved sites** | **Pars. Inf. ^c^** | **Pars. Inf.% ^d^** | **RI^f^** |
| --- | --- | --- | --- | --- | --- | --- | --- |
| 1 | *trnR^UCU^-atpA* | 220 | 379 | 215 | 54 | 20.1 | 0.83 |
| 2 | *trnK^UUU^-rps16* | 586 | 950 | 267 | 44 | 14.1 | 0.74 |
| 3 | *ndhG-ndhI* | 384 | 472 | 335 | 50 | 13.0 | 0.95 |
| 4 | *rps15-ycf1* | 430 | 586 | 410 | 52 | 11.3 | 0.84 |
| 5 | *trnQ^UUG^-psbK* | 391 | 408 | 319 | 32 | 9.1 | 0.92 |
| 6 | *psaC-ndhE* | 288 | 308 | 224 | 22 | 8.9 | 0.83 |
| 7 | *psaJ-rpl33* | 543 | 611 | 467 | 42 | 8.3 | 0.96 |
| 8 | *rps18-rpl20* | 268 | 342 | 277 | 23 | 7.7 | 1.00 |
| 9 | *atpH-atpI* | 758 | 1215 | 955 | 69 | 6.7 | 0.9 |
| 10 | *rps4-trnT^UGU^* | 353 | 371 | 303 | 13 | 4.1 | 1.00 |

a: Length: refers to sequence length in *L.fauriei*; b: Aligned length: refers to the alignment of seven Myrtales species considered in the comparative analysis (see Materials and Methods); c: Number of parsimony informative sites; d: Percentage of parsimony informative sites; e: CI- Consistency Index; f: RI-Retention Index; g: SI- Sequence Identity.
