# Supplementary material for: The Complete Plastid Genome of Lagerstroemia fauriei and Loss of rpl2 Intron from Lagerstroemia (Lythraceae)
Source: PLoS One. 2016 Mar 7;11(3):e0150752. doi: 10.1371/journal.pone.0150752 (PMC4780714; doi:10.1371/journal.pone.0150752)
Supplement: S4 Table — (DOCX) [file pone.0150752.s009.docx]

**S4 Table. Divided genes (longer than 1.5kb) into short regions and their parsimony-informative distribution**

| **No.** | **Region** | **Aligned length (bp)^a^** | **Conserved sites** | **Pars. Inf.^b^** | **Pars. Inf.%^c^** | **RI^d^** |
| --- | --- | --- | --- | --- | --- | --- |
| atpA | 0.0 - 1.0 kb | 1000 | 928 | 24 | 2.59 | 0.92 |
|  | **0.5 - 1.5 kb** | **1001** | **910** | **25** | **2.75** | **0.82** |
|  | 1.0 - 1.5 kp | 525 | 464 | 11 | 2.37 | 0.92 |
| matK | **0.0 -1.0 kb** | **1000** | **812** | **65** | **8.00** | **0.89** |
|  | 0.5 -1.5 kb | 1001 | 811 | 64 | 7.89 | 0.91 |
|  | 1.5 -1.6 kp | 594 | 484 | 33 | 6.82 | 0.97 |
| ndhD | 0.0 - 1.0 kb | 1000 | 869 | 37 | 4.26 | 0.90 |
|  | **0.5-1.5 kb** | **1001** | **866** | **45** | **5.20** | **0.94** |
|  | 1.5 -1.6 kp | 591 | 511 | 25 | 4.89 | 0.97 |
| nahF | 0.0 - 1.0 kb | 1000 | 884 | 35 | 3.96 | 0.95 |
|  | 0.5 -1.5 kb | 1001 | 871 | 39 | 4.48 | 0.84 |
|  | **1.5 - 2.3 kp** | **1344** | **1078** | **78** | **7.24** | **0.77** |
| psaA | 0.0 - 1.0 kb | 1000 | 948 | 14 | 1.48 | 0.93 |
|  | 0.5 -1.5 kb | 1001 | 948 | 16 | 1.69 | 0.85 |
|  | **1.0 - 2.0 kb** | **1001** | **935** | **21** | **2.25** | **0.82** |
|  | 1.5 -2.3 kp | 754 | 709 | 12 | 1.69 | 0.87 |
| psaB | 0.0 - 1.0 kb | 1000 | 900 | 22 | 2.44 | 0.74 |
|  | 0.5 -1.5 kb | 1001 | 873 | 24 | 2.75 | 0.83 |
|  | 1.0 - 2.0 kb | 1001 | 885 | 23 | 2.60 | 0.84 |
|  | **1.5 - 2.2 kb** | **706** | **634** | **19** | **3.00** | **0.83** |
| rpoB | 0.0 - 1.0 kb | 1000 | 944 | 13 | 1.38 | 0.82 |
|  | 0.5 -1.5 kb | 1001 | 933 | 18 | 1.93 | 0.83 |
|  | 1.0 - 2.0 kb | 1001 | 914 | 23 | 2.52 | 0.89 |
|  | **1.5 - 2.5 kb** | **1001** | **907** | **28** | **3.09** | **0.97** |
|  | 2.0 - 3.2 kb | 1229 | 1120 | 32 | 2.86 | 0.89 |
| rpoC1 | 0.0 - 1.0 kb | 1000 | 921 | 18 | 1.95 | 1.00 |
|  | 0.5 -1.5 kb | 1001 | 913 | 15 | 1.64 | 1.00 |
|  | **1.0 - 2.1 kb** | **1074** | **979** | **24** | **2.45** | **0.96** |
| rpoC2 | 0.0 - 1.0 kb | 1000 | 927 | 21 | 2.27 | 0.91 |
|  | 0.5 -1.5 kb | 1001 | 910 | 27 | 2.97 | 0.93 |
|  | **1.0 - 2.0 kb** | **1001** | **845** | **55** | **6.51** | **0.81** |
|  | 1.5 - 2.5 kb | 1001 | 833 | 53 | 6.36 | 0.78 |
|  | 2.0 - 3.0 kb | 1001 | 845 | 33 | 3.91 | 0.78 |
|  | 2.5 - 3.5 kb | 1001 | 837 | 45 | 5.38 | 0.84 |
|  | 3.0 - 4.2 kb | 1201 | 1047 | 50 | 4.78 | 0.88 |
| ycf1 | 0.0 - 1.0 kb | 1000 | 784 | 8 | 1.02 | 1.00 |
|  | 0.5 -1.5 kb | 1001 | 729 | 19 | 2.61 | 0.91 |
|  | 1.0 - 2.0 kb | 1001 | 687 | 62 | 9.02 | 0.72 |
|  | 1.5 - 2.5 kb | 1001 | 757 | 72 | 9.51 | 0.62 |
|  | 2.0 - 3.0 kb | 1001 | 712 | 55 | 7.72 | 0.61 |
|  | 2.5 - 3.5 kb | 1001 | 622 | 51 | 8.20 | 0.67 |
|  | 3.0 - 4.0 kb | 1001 | 676 | 41 | 6.07 | 0.71 |
|  | 3.5 - 4.5 kb | 1001 | 700 | 55 | 7.86 | 0.65 |
|  | 4.0 - 5.0 kb | 1001 | 634 | 78 | 12.30 | 0.69 |
|  | 4.5 - 5.5 kb | 1001 | 602 | 79 | 13.12 | 0.82 |
|  | **5.0 - 6.0 kb** | **1001** | **490** | **77** | **15.71** | **0.83** |
|  | 5.5 - 6.5 kb | 1001 | 438 | 64 | 14.61 | 0.70 |
|  | 6.0 - 7.4 kp | 1410 | 620 | 74 | 11.94 | 0.63 |
| ycf2 | 0.0 - 1.0 kb | 1000 | 810 | 6 | 0.74 | 0.90 |
|  | 0.5 -1.5 kb | 1001 | 715 | 8 | 1.12 | 0.83 |
|  | 1.0 - 2.0 kb | 1001 | 868 | 7 | 0.81 | 1.00 |
|  | 1.5 - 2.5 kb | 1001 | 945 | 12 | 1.27 | 0.86 |
|  | 2.0 - 3.0 kb | 1001 | 930 | 10 | 1.08 | 0.83 |
|  | 2.5 - 3.5 kb | 1001 | 921 | 9 | 0.98 | 1.00 |
|  | 3.0 - 4.0 kb | 1001 | 929 | 9 | 0.97 | 0.91 |
|  | 3.5 - 4.5 kb | 1001 | 883 | 12 | 1.36 | 0.87 |
|  | **4.0 - 5.0 kb** | **1001** | **773** | **13** | **1.68** | **0.86** |
|  | 4.5 - 5.5 kb | 1001 | 794 | 8 | 1.01 | 0.78 |
|  | 5.0 - 6.0 kb | 1001 | 880 | 8 | 0.91 | 1.00 |
|  | 5.5 - 6.5 kb | 1001 | 893 | 6 | 0.67 | 1.00 |
|  | 6.0 - 7.0 kb | 1001 | 951 | 2 | 0.21 | 1.00 |
|  | 6.5 - 7.5 kb | 1001 | 914 | 1 | 0.11 | 1.00 |
|  | 7.0 - 8.1 kp | 1062 | 877 | 3 | 0.34 | 1.00 |

The bold font represents the most variable fragment within gene.

a: Aligned length: refers to the alignment of seven Myrtales species considered in the comparative analysis (see Materials and Methods);

b: Number of parsimony informative sites;

c: Percentage of parsimony informative sites;

d: RI-Ensemble retention index.
